# The first Swedish H1N2 swine influenza virus isolate represents an uncommon reassortant

**DOI:** 10.1186/1743-422X-6-180

**Published:** 2009-10-28

**Authors:** Ádám Bálint, Giorgi Metreveli, Frederik Widén, Siamak Zohari, Mikael Berg, Mats Isaksson, Lena HM Renström, Per Wallgren, Sándor Belák, Thomas Segall, István Kiss

**Affiliations:** 1 National Veterinary Institute, (SVA), Ulls väg 2B, SE-751 89 Uppsala, Sweden; 2 Department of Virology, Central Agricultural Office, Veterinary Diagnostic Directorate, Tábornok u2, H-1149 Budapest, Hungary; 3 Swedish University of Agricultural Sciences (SLU), Ulls väg 2B, SE-751 89 Uppsala, Sweden; 4 Department of Microbiology, Central Agricultural Office, Veterinary Diagnostic Directorate, Bornemissza u 3-7, H-4031 Debrecen, Hungary

## Abstract

The European swine influenza viruses (SIVs) show considerable diversity comprising different types of H1N1, H3N2, and H1N2 strains. The intensifying full genome sequencing efforts reveal further reassortants within these subtypes. Here we report the identification of an uncommon reassortant variant of H1N2 subtype influenza virus isolated from a pig in a multisite herd where H1N2 swine influenza was diagnosed for the first time in Sweden during the winter of 2008-2009. The majority of the European H1N2 swine influenza viruses described so far possess haemagglutinin (HA) of the human-like H1N2 SIV viruses and the neuraminidase (NA) of either the European H1N2 or H3N2 SIV-like viruses. The Swedish isolate has an avian-like SIV HA and a H3N2 SIV-like NA, which is phylogenetically more closely related to H3N2 SIV NAs from isolates collected in the early '80s than to the NA of H3N2 origin of the H1N2 viruses isolated during the last decade, as depicted by some German strains, indicative of independent acquisition of the NA genes for these two types of reassortants. The internal genes proved to be entirely of avian-like SIV H1N1 origin. The prevalence of this SIV variant in pig populations needs to be determined, as well as the suitability of the routinely used laboratory reagents to analyze this strain.

The description of this H1N2 SIV adds further information to influenza epidemiology and supports the necessity of surveillance for influenza viruses in pigs.

## Findings

Swine influenza viruses (SIVs) have a world-wide distribution, and may cause respiratory disease in pigs of rapid and dramatic onset. The causative agent belongs to type A influenza viruses within the *Orthomyxoviridae *family. In North America, classical swine H1N1 viruses, triple-reassortant H3N2 viruses, possessing genes of classical swine H1N1, North American avian, and human H3N2 viruses, and different lineages of H1N1 as well as H1N2 viruses generated by reassortations from the afore-mentioned ones co-circulate. In Asia, the situation is even more complex, since in addition to the above viruses, human H3N2 and Eurasian avian-like H1N1 SIVs are present [1,2]. In Europe, an avian-like H1N1 virus, which was first detected in 1979 in Belgium, replaced the classical swine H1N1 viruses and became predominating [3]. These avian-like SIVs reassorted with human H3N2 viruses, and gave rise to human-like H3N2 SIVs, first detected in the mid eighties in Italy [4]. In the same period of time, H1N2 SIVs comprising HA of classical swine H1N1 and human-like NA from swine H3N2 viruses were isolated in France [5-7]. These reassortants have not become widespread among the European pig population and unfortunately, no nucleotide sequence data are available in the GenBank regarding these isolates. In 1994, a further reassortant variant was identified in the UK, comprising haemagglutinin (HA) and neuraminidase (NA) genes of human origin (human-like H1N2 viruses), [8,9]. Since then, the HA of these viruses evolved independently from their ancestor human viruses [10].

Recently, "novel" reassortant H1N2 SIVs were isolated in Germany (A/sw/Cloppenburg/IDT4777/05 and A/sw/Dötlingen/IDT4735/05) having a mixture of the characteristics of porcine H1N2 and H3N2 viruses [11]. Serological investigations suggested that these H1N2 viruses should be antigenically different from the first isolated German H1N2 SIV strain, A/swine/Bakum/1832/2000 [12], which was considered as a typical representative of German H1N2 SIVs. The molecular investigations confirmed this assumption, since indeed, the novel German H1N2 strains contained a H3N2 SIV-like NA represented by A/swine/Ghent/1/1984 (H3N2), and not an "established" European H1N2 NA represented by and supposedly evolved from A/swine/Scotland/41044/1994 (H1N2); [11]. Their HA however, grouped together with other European human-like H1N2 HAs. Therefore, a reassortment event between the prevalent European H1N2 and H3N2 SIVs was suggested in the background of the emergence of the new H1N2 viruses.

Showing the complexity of reassortations, the same study noticed a "unique" Italian virus, A/swine/Italy/2064/1999 (H1N2), which had an avian-like H1 and a European SIV-like H1N2 NA. A GenBank search revealed only one additional strain with similar composition, A/swine/Germany/SEk1178/2000 (H1N2) of northern Germany origin.

The A/swine/Sweden/1021/2009 (H1N2) virus was isolated as part of the diagnostic investigation of a multisite pig herd (sow pool) with 4,000 sows affected with severe clinical signs of respiratory disease among growers during the winter of 2008-2009. The herd was centrally located in Sweden, 400 km from the southern coast line and 200 km from the western coast line. Nevertheless, this was the first demonstration of H1N2 in pigs in Sweden [13]. However, the location of the herd makes it less likely to believe that this was the true index case of H1N2 in Sweden, and the significance of the herd size for development of clinical signs will be scrutinized. According to a national serologic screening carried out in 2006, pigs in Sweden were free from H1N2 at that time. Previously, influenza H1N1 infected the Swedish pig population for the first time during the winter 1982-1983 causing severe clinical signs in pigs of all ages at that time [14]. Thereafter the clinical signs of H1N1 have declined. H3N2 established itself less dramatically than H1N1, and the index year therefore remains unknown. A national serologic screening carried out in 1999 demonstrated that H3N2 was well established in the country.

Since clinical manifestation of swine influenza is rare in Sweden, and SIV strains are of particular concern due to the novel human H1N1 epidemic, the virus was isolated in primary swine kidney cells based on standard protocols and the isolate was subjected to full genome sequencing and sequence analysis as described earlier [15]. Briefly, the coding sequences were amplified in one piece (for HA, M, NA, and NS genes) or in two fragments for the rest of the genes, and sequenced afterwards supported by additional sequencing primers in case it was necessary. For the phylogenetic analyses nucleotide sequences were collected based on the BLAST search results of the obtained sequences, additional sequences were obtained from the Influenza Virus Resource platform of NCBI , plus, if still missed, sequences of isolates appearing in the related publications were specifically searched for. Sequence assembly, multiple alignment and alignment trimming were performed with the CLC Main Workbench 5.0.2. (CLC bio A/S, Aarhus, Denmark). Distance based neighbor joining and character based maximum parsimony phylogenetic trees were generated using the Molecular Evolutionary Genetics Analysis (MEGA) software v.4.0. [16], with 1000 bootstrap replicates. For the neighbor-joining trees, the Maximum Composite Likelihood method was used. Other models were also tested which showed similar topologies.

**Figure 1:**
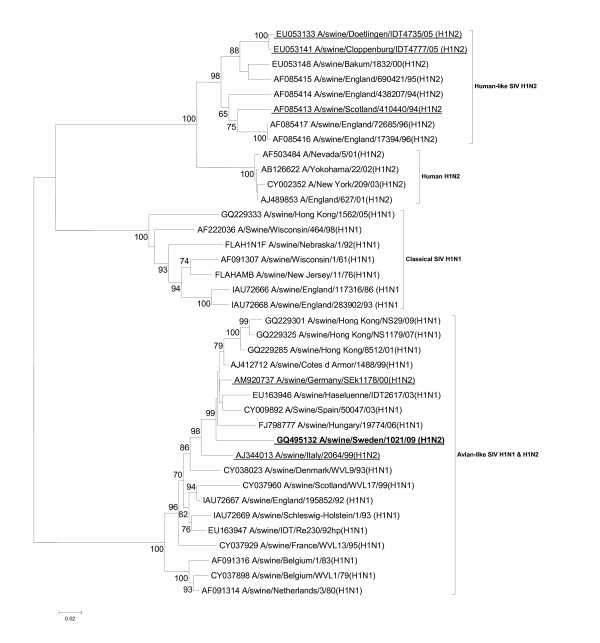
**Evolutionary relationships of the HA gene of A/swine/Sweden/1021/09 (H1N2) compared to genetically related influenza viruses**. The phylogenetic tree was generated by the neighbor-joining method. Bootstrap values of 1000 resamplings in per cent are indicated at key nodes. The Swedish virus is highlighted with bold letters and the representative isolates referred to in Table 1 are underlined.

**Figure 2:**
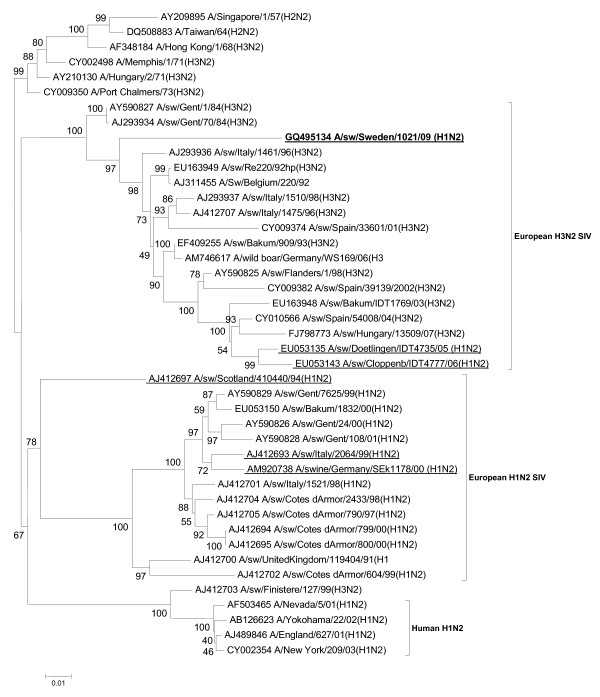
**Evolutionary relationships of the NA gene of A/swine/Sweden/1021/09 (H1N2) compared to genetically related influenza viruses**. The phylogenetic tree was generated by the neighbor-joining method. Bootstrap values of 1000 resamplings in per cent are indicated at key nodes. The Swedish virus is highlighted with bold letters and the representative isolates referred to in Table 1 are underlined.

The subtyping of the isolate by the analysis of nucleotide homology revealed its H1N2 nature. The phylogenetic investigations clarified that the H1 belonged to the avian-like H1N1 SIVs, which is in contrast to the "established" H1N2 SIVs in Europe (Figure 1). The two "unique" viruses from Italy and northern Germany grouped together with the Swedish isolate. The NA of the Swedish virus was similar to those of the "novel" A/sw/Cloppenburg/IDT4777/05 and A/sw/Dötlingen/IDT4735/05 isolates by grouping together with European H3N2 SIVs (Figure 2). However, the Swedish strain showed higher nucleotide identity to the A/swine/Gent/1/84 (H3N2) (89.39%) than to the German strains (84.5 and 85%, respectively), which is reflected also by their phylogenetic positions. These data indicate that the Swedish strain obtained its N2 gene independently of the German strains. The summary of the surface glycoprotein composition of the European H1N2 SIVs described so far is presented in Table 1.

**Table 1:** Schematic presentation of the HA and NA composition of European H1N2 SIV isolates identified so far

**Type**	**Representative(s)**	**HAH1**	**NAN2**
"early"	A/swine/Scotland/410440/94	Human **H1**	Human H3**N2**

"established" European	A/swine/Bakum/1832/00	Human-like **H1**N2	European SIV-like H1**N2**

"unique" German, Italian	A/swine/Germany/SEk1178/2000 A/swine/Italy/2064/99	Avian-like SIV **H1**N1	European SIV-like H1**N2**

"novel" German	A/swine/Dötlingen/IDT4735/05 A/swineburg/Cloppenburg/IDT4777/05	Human-like **H1**N2	European SIV-like H3**N2**

Swedish	A/swine/Sweden/1021/09	Avian-like SIV **H1**N1	European SIV-like H3**N2**

The "donor" segments are indicated with bold characters.

The internal genes proved to be of avian-like SIV H1N1 origin supported both by phylogenetic analyses and the relevant markers presented by Dunham et al., [1], although one marker assigned to PB1 and NS1 each was shared with avian viruses (data not shown), reflecting the ancestors of these inner genes. It was noticed in the case of each phylogenetic tree, however, that the Swedish isolate sit on a rather long branch next to its closest relatives regardless of the method used for phylogeny. This phenomenon reflects the lack of available swine influenza sequences from Scandinavia in the GenBank and about SIVs of similar characteristics. The accumulation of further data of the relevant type should improve the situation. The PB1-F2 protein encoded by an alternative ORF of PB1 [17] proved to be truncated in the case of the Swedish virus due to possessing a stop codon after the eleventh amino acid, characterizing a minor proportion of SIVs [18].

Uniformly with the prevalent European SIVs, the M2 gene of the Swedish virus possessed the S31N substitution that is indicative of amantadine resistance [19].

In summary, the characterised Swedish isolate possessing avian-like SIV H1N1 HA and European H3N2 SIV-like NA represents an uncommon type of H1N2 SIV reassortant virus and further, its NA appears to be obtained by a reassortment independently of the recent German H1N2 SIVs, otherwise similar in composition. The origin, antigenic characteristics of the strain and its prevalence in Sweden are yet unknown and are the subjects of further investigations.

The presented findings support the observations concerning the continuous reassortment process of SIVs, resulting in repeated and independent emergence of certain HA/NA combinations in pigs, and also the need for the systematic surveillance of influenza in swine to reveal such events and identify these strains.

Nucleotide sequence accession numbers

Nucleotide sequences from the A/swine/Sweden/1021/09 (H1N2) isolate have been submitted to GenBank with accession numbers GQ495129-GQ495136.

## Competing interests

The authors declare that they have no competing interests.

## Authors' contributions

AB and IK performed sequence analyses, alignments, phylogenies, interpretation of data, carried out identification of viruses and wrote the manuscript. GM carried out PCR and sequencing reactions and optimized protocols. SZ, MB, MI, and SB contributed to the interpretation of the findings and revised the manuscript. FW, LR, TS and PW obtained the clinical samples, organized sample processing and revised the manuscript. All authors read and approved the final manuscript.
